# Sintering Nano-Silver Paste by Resistive Joule Heating Process for 2G HTS Tape Joints

**DOI:** 10.3390/ma15041571

**Published:** 2022-02-19

**Authors:** Chia-Ming Yang, Yu-Chuan Chang, Chi-Lei Chang, In-Gann Chen

**Affiliations:** Department of Materials Science and Engineering, National Cheng-Kung University, Tainan 701, Taiwan; lf2killer@gmail.com (C.-M.Y.); eric77772277@gmail.com (Y.-C.C.); zachary880127@gmail.com (C.-L.C.)

**Keywords:** 2G HTS tape, solder joint, nano-silver paste, resistive Joule heating, specific resistance

## Abstract

Developing a joining technology for 2G HTS tapes without significantly reducing their superconducting property is crucial for numerous applications (MRI, motor/generator, power transmission, etc.). In this study, low sintering temperature (~230 °C) nano-silver paste was used as solder to join two 2G HTS tapes. In addition, two heating methods, i.e., furnace heating (heat flux outside-in) and resistive Joule heating (heat flux inside-out), were studied. This study indicates that the heat flux from internal by resistive Joule heating method shows less deteriorating impact to the 2G RE-Ba-Cu-O tape (RE: rare earth element) during the sintering process with the best specific resistance of 0.074 μΩ∙cm^2^ and *I_c_* retention percentage of 99% (i.e., *I_c_* reduced from 100 A before joining to 99 A after joining). This study indicates that nano-silver paste together with resistive Joule heating can possibly be used as soldering materials to join 2G HTS tapes.

## 1. Introduction

RE-Ba-Cu-O (RE: rare earth element) superconductors show high critical current density (*J_c_*) that can be used for high field magnets (>20 T) [1,2,3,4]. However, RE-Ba-Cu-O superconductors suffer from the weak-link effect, indicating that defects such as high angle grain boundaries could block the superconducting current and reduce its *J_c_* exponentially as current increase. For this reason, commercial RE-Ba-Cu-O superconductor wire made of multi-layer epitaxial film, called 2G HTS (2nd high temperature superconductor) tapes, has been created [5,6,7].

However, due to the limitation in producing continuous epitaxy microstructure over a long length (>1000 m), the length of 2G HTS tapes produced is still significantly shorter than most strong magnetic field applications (e.g., nuclear magnetic resonance, motor/generator, fusion magnets, etc.) required (several kilometers). Therefore, it is necessary to develop a joining technology for the 2G HTS tapes without significantly reducing its superconducting properties, such as critical current density and specific resistance of the joint. According to previous studies, there are three types of joining processes: the solder joint method, the diffusion joint method, and the superconducting joint method. The solder joint method uses low melting point solder, such as Sn-alloy, to join (weld) the 2G HTS tapes; the specific resistance of the joint is about 0.02–1 μΩ∙cm^2^ [8,9,10,11,12,13]. The diffusion joint method requires a relatively high temperature and compression pressure to cause inter-diffusion at the joint, such as Cu or Ag of the protective layer of 2G HTS tape, with the specific resistance of about 0.067 μΩ∙cm^2^ [13,14]. The superconducting joint method uses superconductive material to join the 2G HTS tapes, with specific resistance of <10^−6^ μΩ∙cm^2^ [15,16,17,18,19]. Even though the superconducting joint method shows the lowest specific resistance at the joint, the retention superconducting properties are unstable after the high treatment temperature (>600 °C), which may cause damage of the epitaxy microstructure and oxygen stoichiometry [20,21]. To resolve this problem, a post oxygen annealing process was needed after the superconductive joint method. This complicated manufacturing process was not conducive to practical applications; therefore, most studies have continued to focus on the solder joint method.

For the solder joint method, Huang et al. [9] used Sn_36_Pb_37_ as the solder for the 2G HTS tape joining and sintered at 190 °C with a bonding pressure of 10MPa; the specific resistance of the joint was about 0.034 μΩ∙cm^2^. He also mentioned that if the thickness of the copper protective layer of the wire is reduced (from 20 μm to 5 μm) through a proper etching method, the specific resistance of the joint would be reduced to about 0.025–0.027 μΩ∙cm^2^ without affecting the superconducting properties of the 2G HTS tape. In addition, Balashov et al. [10] also used Pb_39_Sn_61_ as the bonding solder, and immersed the 2G HTS tapes in a molten solder bath at 200 °C. A relatively lower specific resistivity was obtained reproducibly by this method, in the range of 0.01–0.015 μΩ∙cm^2^.

Nishio et al. obtained indium foil as the joint material and achieved the specific resistance of 0.025 μΩ∙cm^2^ with a pressure of 100MPa at low heat treatment temperatures of 90~140 °C [11]. Ito et al. also used indium foil as the joint material and welded it by the ultrasonic method [12], in attempting to shorten the joining process time using this method (<0.1 s); the specific resistance was about 0.031~0.032 μΩ∙cm^2^. In comparison with that of PbSn alloy, the indium foil and indium tin alloy showed low thermal stability, insufficient strength, and high brittleness. Nakanishi et al. reported briefly on using nano-silver paste for the 2G HTS tape joint with a specific resistance of ~0.048 μΩ∙cm^2^ [13].

In general, regardless of the joint material, the solder joint process needs a sintering temperature (>200 °C) to reach low specific resistance with the order of 10^−2^ μΩ∙cm^2^. However, further increasing the sinter temperature will decrease the retention critical current density of the 2G HTS tapes due to the release of oxygen of the RE-Ba-Cu-O [13], and even oxidize the protective/stabilizer layers (Cu). Therefore, the precise control of the sintering temperature is a key parameter for the joining process.

In this study, low sintering temperature (~230 °C) nano-silver paste was used as solder to join two 2G HTS tapes. In addition, two heating methods, i.e., furnace heating (heat flux outside-in) and resistive Joule heating (heat flux inside-out), were studied. This study uses the resistive Joule heating to generate localized heating internally on the soldering materials [22,23,24]. This inside-out heating flux profile will avoid overheating the RE-Ba-Cu-O film located outside the joint that may possibly retain higher superconducting properties. Moreover, the sintered nano-silver paste can improve the heat dissipation of the joint (thermal conductivity >200 W/m∙K) [25] and also show high thermal stability to avoid forming cracks and IMC (intermetallic compound).

## 2. Materials and Methods

### 2.1. Preparation of Tape-to-Tape Joints

**Figure 1:**
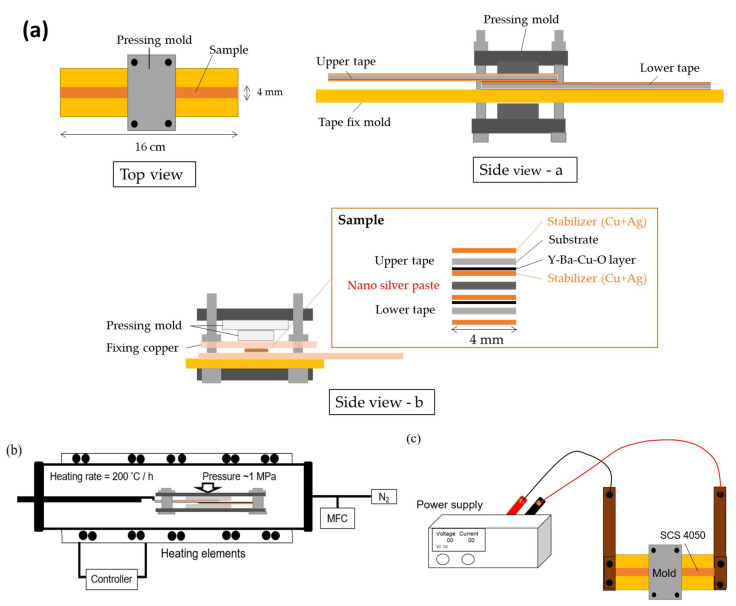
Schematic diagram of the experimental process: (**a**) the joint structure: the tape (SCS 4050)-solder (nano-silver paste)-tape (SCS 4050) joint, with the area of 5 × 4 mm^2^, was fixed by a special mold with compressive stress of 1–5 MPa; (**b**) the furnace heating process: joining samples fixed by the mold with the pressure of 1 MPa were put in the tube furnace; (**c**) the resistive Joule heating process: the samples were fixed by the mold with the pressure of 5 MPa, and electrodes were directly installed both ends of the HTS tapes and applied current.

The commercial 2G HTS tape (SCS4050, Superpower Inc., Schenectady, NY, USA), with the epitaxy microstructure of Y-Ba-Cu-O thin film (~1 μm thickness)/stabilizer (protect) layer Ag (2 μm thickness)/stabilizer (protect) layer Cu (20 μm thickness), was used in this study, as shown in Figure 1a. To prepare the joining samples, first the 2G HTS tapes were cut into 10 cm pieces and their surfaces were cleaned with alcohol. Then, the I–V curves at 77 K of these samples were measured to confirm their superconducting critical current (*I_c_*). After the measurement, the homemade nano-silver paste was screen printed on the joint area (5 × 4 mm^2^) of a HTS tape with another HTS tape covering it. The homemade silver paste was composed of the α-terpineol (Sigma–Aldrich, Darmstadt, Germany, >96%) and silver nanoparticles (70 wt%) with a particle size of 10–50 nm. Details including the synthesis of the silver nanoparticles can be found in past studies [26]. Finally, the whole tape-solder (nano-silver paste)-tape joint structure was fixed by a special mold, as shown in Figure 1a.

### 2.2. Heat Treatment of the Tape-Solder (Nano-Silver Paste)-Tape Joint Sample

Two heating process were conducted in this study: the furnace heating process and the resistive Joule heating process. For the furnace heating process, as shown in Figure 1b, the tape-solder (nano-silver paste)-tape joint samples were fixed by the compressive mold with the pressure of 1 MPa and put into the tube furnace and heated to 180–250 °C for 1 h at the rate of 200 °C/h in N_2_ atmosphere and then furnace cooled to 25 °C. For the resistive Joule heating process, the samples were fixed by the mold with the pressure of 5 MPa, with electrodes directly installed in both ends of the HTS tapes; then, a current of 100–150 A was applied by a power supply (Aglient 6681A, Agilent Technologies, Santa Clara, CA, USA), as shown in Figure 1c. Considering safety, the current was increased step by step to 100–150 A at the rate of 10 A/min. The temperature of the samples was inspected by the thermo couples (K-Type, TES-1314a, TES Electrical Electronic Corp., Taipei, Taiwan).

### 2.3. Analysis

The four-point method (power supply: Aglient 6681A, voltage meter: Keithley 195A, Cleveland, OH, USA) was used to measure the I-V curve of the 2G HTS tapes and joining samples at both room temperature and 77 K (liquid nitrogen). The interval of the voltage probe was 5 cm.

## 3. Results

### 3.1. The Furnace Heating Process

**Figure 2:**
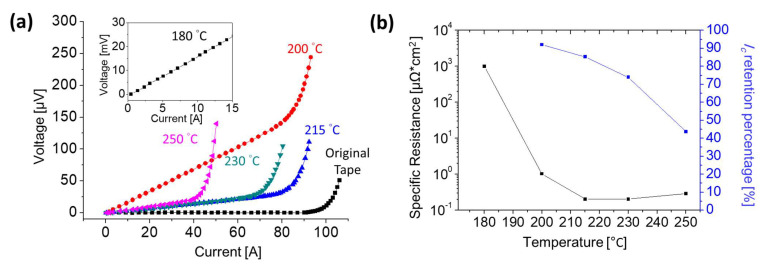
(**a**) The I-V curve results of 2G HTS tape joining samples measured at 77 K under different heating temperatures. Due to the joint large resistance, the I-V curve of the 180 °C heating is displayed in the inset; the original tape is the HTS tape before heating at 215 °C. (**b**) The *I_c_* percentage and specific resistance of the 2G HTS tape joint samples heated at different temperatures.

Figure 2a shows the I-V curve at 77 K of the original HTS tape (before heating) and the joining samples heated at different temperatures in the tube furnace. Due to the relatively large joint resistance (the slope of the I-V curve), the I-V curve of the 180 °C heating sample is shown in the inset. It was clearly observed that the original tape (the tape before heating at 215 °C) shows the better resistance (no joint) and there was no significant difference in resistance (joint resistance) of the samples after heating at 215 °C, 230 °C, and 250 °C. Moreover, the critical current (*I_c_*) decreased and the heating temperature increased.

As had already been established, for an HTS tape without joint, the electric field (E-field) criterion used for *I_c_* was defined as 1 μV/cm. However, for the HTS tapes joining sample, the *I-V* curve of a superconductor could be expressed by the formula: *V*(*I*) = *R_j_* × *I* + *V_c_* (*I*/ *I_c_*)*^n^* [8]; where *R_j_* is the joint resistance; *V_c_* is the voltage referenced to the E-field criterion (1 μV/cm); *I_c_* is the critical current; and *n* is the index. The joint resistance and *I_c_* of all samples can be obtained by this formula and Table 1 lists the analysis results of the different heating temperature samples (Furnace 1–5). In order to avoid the error caused by the differences of the original HTS tape, the *I_c_* retention percentage (*I_c_* after joining/*I_c_* before joining) was used to compare superconductivity of the joining samples. Figure 2b shows the *I_c_* retention percentage and the specific resistance of all samples. Note that the *I_c_* retention percentage was only 44% for the sample heated at 250 °C, which meant that superconductivity of the sample was severely affected by the heating process. It was found that the *I_c_* retention percentage increased with the decrease of the heat treatment temperature, and the *I_c_* retention percentage of the sample heated at 200 °C was about 90%. Due to the extremely high joint resistance, the *I_c_* retention percentage of the sample heated at 180 °C could not be analyzed.

Compared with the *I_c_* retention percentage, the result of the specific resistance (resistance × joint area) at different heating temperature was more complicated. First, the specific resistance of the samples after heating at 215 °C and 230 °C was almost the same. When the heating temperature increased to 250 °C, the specific resistance increased slightly due to the oxidation of the copper layer. As the heating temperature decreased, the specific resistance increased sharply; it was 1 μΩ∙cm^2^ for the 200 °C heated sample and 948 μΩ∙cm^2^ for the 180 °C heated sample. The TGA (thermogravimetric analysis) results of the nano-silver paste showed that the decomposition temperature of the nano-particle protective agent was about 180–200 °C, indicating that the sintering temperature of it should be larger than 200 °C. This was why the specific resistance values of the 180 °C and 200 °C samples were larger than those of other samples. Moreover, the similar values of specific resistance of the 215 °C, 230 °C, and 250 °C heated samples suggested that the higher heating temperature did not help the sintering of the nano-silver paste.

### 3.2. The Resistive Joule Heating Process

From the furnace heating results, it was concluded that the most suitable heating temperature for the nano-silver paste joining process was 200–215 °C, which showed the best values in terms of the specific resistance and *I_c_* retention percentage. Therefore, the next step was to explore how to use electric current to achieve local resistive Joule heating to a temperature 200–215 °C. For the resistive Joule heating process, the resistance of a joint area should be greater than those of other parts of the circuit. From our measurement, the resistance of the joint (3–10 mΩ/cm) was at least 10 times greater than that of the tapes (<0.3 mΩ/cm); otherwise, the heating phenomenon would spread to the entire tape (not just at joint).

**Figure 3:**
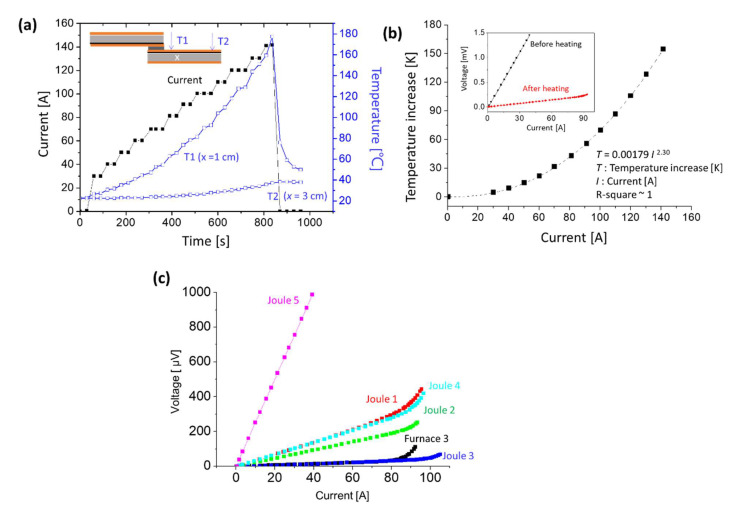
(**a**) Temperatures of T1 and T2 thermocouples, which were set at 1 cm and 3 cm away from the joint as shown in the inset. When the input current reached 140 A, the T1 and T2 thermocouple showed the temperature of 180 °C and 40 °C, respectively. (**b**) The temperature increase of the T1 thermocouple versus the current. It was fitted by the power law (*T* = 0.00179 *I* ^2.30^), with the R-square ~1, which agreed with the Joule law. The inset shows the I-V curves of the sample before and after resistive Joule heating. It was observed that the resistance decreased from 36 μΩ to 2.3~3.4 μΩ at room temperature, indicating that the resistive Joule heating process was effective. (**c**) The I-V curve results of 2G HTS tape joint samples measured at 77 K under different Joule heating temperatures.

In order to observe the temperature change during the resistive Joule heating process, two thermocouples were set at 1 cm and 3 cm away from the joint; their temperatures are shown in Figure 3a. Both thermocouples showed temperature of 30 °C before current inputting into the joint, and the temperature became higher in both T1 and T2 as the higher inputting current. At the same time, the temperature difference between T1 and T2 became larger as the input current increased. Finally, when the current reached 140 A, the T1 thermocouple had reached 180 °C and the T2 thermocouple was only about 40 °C. This result indicates that due to the large resistance of the joint, the heating phenomenon can be concentrated on it. In addition, the relationship between the temperature rise of the T1 thermocouple and the current is shown in Figure 3b, which can be fitted by the power law (*T* = 0.00179 *I* ^2.30^), with the R-square ~1. The R-square value indicates how well observed outcomes are replicated by the model. The bigger R-square value signifies better fitting quality, and the value will be in the range of 0~1. This result implied our heating process was stable without the abnormal heat flowing. For the typical Joule law, the temperature rise of the T1 was related to the square of current, and our fitting results were roughly close to it, although the heat loss in the environments may cause some slight errors. Figure 3b shows the I-V curves of the sample before and after the resistive Joule heating process. It was observed that the resistance decreased from 36 μΩ to 2.3~3.4 μΩ at room temperature, indicating that the resistive Joule heating process was effective. The I-V analysis result of this sample (Joule 1) at 77 K was listed in Table 1. Though the specific resistance of the Joule 1 sample was 0.47 μΩ∙cm^2^, which was larger than that of the furnace heating sample (0.2 μΩ∙cm^2^), it is worth noting that the *I_c_* retention percentage of it was 98%, while the furnace heating sample was 85%. These results indicated that the resistive Joule heating did little damage to the HTS tapes.

It was noticed that the T1 thermocouple was located 1 cm away from the joint because of the space limit, which was not the actual temperature of the nano-silver paste (joint). This was why the T1 thermocouple only showed 180 °C for the Joule 1 sample and the nano-silver paste was successfully sintered to obtain a good joint. To find the best process parameters of the resistive Joule heating method, different samples (Joule 2–5) were heated at different temperatures. Their I-V curves and specific resistances are shown in Figure 3c and Table 1. The Joule 5 sample exhibited high specific resistance and could be easily observed in Figure 3c, which meant that the temperature of its joint might be greater than 250 °C (T1 = 228.6 °C), causing oxidation of the copper layer. Although the Joule 3 sample exhibited the specific resistance of 0.074 μΩ∙cm^2^ and *I_c_* retention percentage of 99%, which was better than that of the best sample of the furnace heating method (Furnace 3), most of the resistive Joule heating sample shows specific resistances larger than that of the best sample of the furnace heating method (Furnace 3). However, it was noticed that the *I_c_* of the resistive Joule heating sample was very high, showing a high *I_c_* retention ratio compared with the furnace heating sample.

## 4. Discussion

**Figure 4:**
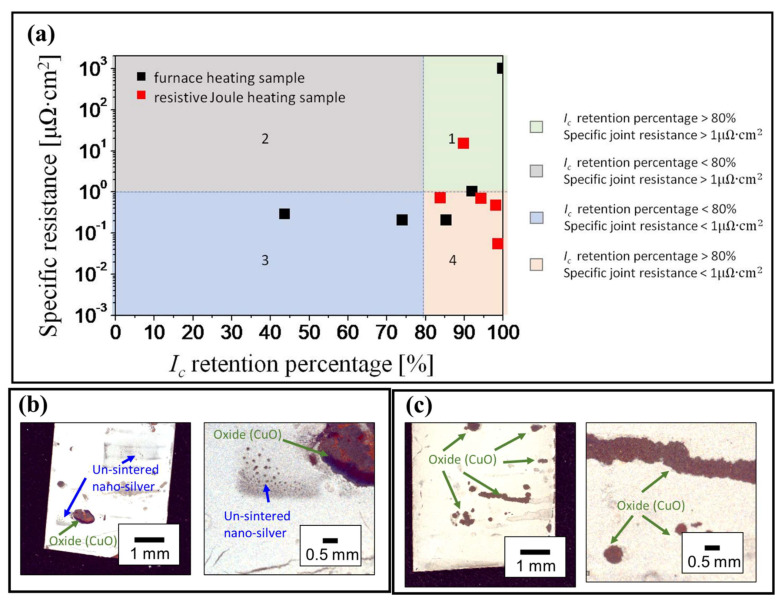
(**a**) The relationship between the *I_c_* retention percentage and specific resistance of samples sintered by furnace heating and resistive Joule heating. Four different blocks are classified, and the boundaries conditions were specific resistance = 1 μΩ∙cm^2^ and *I_c_* retention percentage = 80%. Block 4 shows the better properties samples, which exhibited high *I_c_* retention percentage and low specific resistance. (**b**) The different magnification OM (optical microscope) image of the test sample for the nano-silver paste, heated at 180 °C, which showed dark gray color due to the un-sintered nano-silver particle. A small amount of oxide (CuO) was also observed in the test sample, which was caused by the sintered silver spalling due to the oxidation of the Cu substrate. (**c**) The different magnification OM image of the test sample for the nano-silver paste heated at 250 °C. They show more oxides on the surface than that of the 180 °C heated sample.

Figure 4a shows the *I_c_* retention percentage versus specific resistance of the different furnace heating and resistive Joule heating samples. Most studies had suggested that a good joint required low specific resistance (<1 μΩ∙cm^2^) [27,28,29] and high *I_c_* retention percentage (>80%) [27], shown in Figure 4a with four clearly distinguished blocks. The first block indicates property of high specific resistance (>1 μΩ∙cm^2^) due to insufficient sintering of the nano-silver paste, and shows the obvious feature that some un-sintered nano-silver particle remains dark gray instead of white after the process, as shown in Figure 4b, which was observed in the test sample heated at 180 °C (furnace heating). A small amount of oxide (CuO) was also observed in the test sample, which was caused by the sintered silver spalling due to the oxidation of the Cu substrate. For the furnace heating method, the 180 °C and 200 °C heated samples are no doubt in this second block because removing protective agent should be larger than 200 °C. However, there is only one resistive Joule heating sample in this block, which means that most samples have reached at least 200 °C during the resistive Joule heating process.

The samples in the third show property of low *I_c_* retention percentage (<80%), indicating that their HTS superconductivity was affected by the relative high temperature process and some oxidation has occurred on the tape surface, which was observed in the test sample heated at 250 °C (furnace heating) (Figure 4c). Generally, some studies [9,13] have suggested a heating temperature below 250 °C can avoid the low *I_c_* retention percentage problem of the 2G HTS tape. Our heating temperature was below 250 °C, but two samples are still found in this block. The fourth block is the most suitable block for the joints; most of the resistive Joule heating samples are in this block but only one is the furnace heating sample. The sample in the second block is the worst, that is, the nano-silver paste is not successfully sintered and superconducting properties of the 2G HTS tapes are destroyed; however, this study has not found any sample in this block.

Generally, due to the small thickness of the 2G HTS tape (Cu~20 μm, Ag~2 μm, YBCO~1 μm), its temperature gradient during heating is less than 1 K. However, there is a heat resistance at the interface between the nano-silver paste and the 2G HTS tape, which will affect the heat transfer and cause a temperature difference between them. The temperature jump between the nano-silver paste and the Cu-layer of 2G HTS tape is the interfacial thermal resistance, which is caused by the air gap in the joint and is related to the copper surface roughness and joint quality [30,31]. When the heating process continues, the temperature difference between the nano-silver paste and 2G HTS tape will gradually decrease. In addition, the heated area for the furnace heating method was much larger than that for the resistive Joule heating method. Therefore, the heat flux directions and heated area difference between the furnace heating and resistive Joule heating samples were the main reasons causing different joint performances.

**Figure 5:**
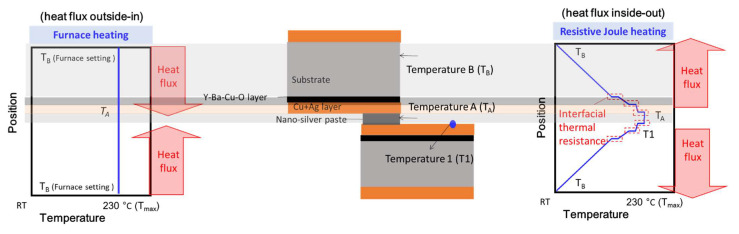
Schematic diagram of the heat flux directions and temperature distribution of the furnace heating and resistive Joule heating process. For furnace heating, the heating rate of the furnace heating method was slow (200 °C/h), so the temperature distribution may be close to steady state and with no obvious temperature gradient. For the resistive Joule heating, the location of the highest temperature will be at the nano-silver paste. Therefore, the temperature of the RE-Ba-Cu-O layer will be lower than that of the nano-silver paste and show less of a deteriorating effect on *I_c_*.

For the furnace heating method (heat flux outside-in), generally, since the heat comes from the furnace, the temperature of the 2G HTS tape surface increases first, and the heat then slowly transfers to the nano-silver paste. However, the heating rate of the furnace heating method was slow (200 °C/h), so the temperature distribution was close to steady state and there was no obvious temperature gradient, as shown in Figure 5. This means that the 2G HTS tapes will continue to deteriorate due to prolonged (>1 h) exposure to temperatures of 230 °C or higher.

For the resistive Joule heating method (heat flux inside-out), as shown in Figure 5, when the current was just inputted to the joint sample, due to the large internal resistance, only the nano-silver paste increased the temperature. If this temperature exceeds 200 °C, the nano-silver paste will begin to sinter. It should be noticed that the current input time should be as small as possible to eliminate the temperature change of the 2G HTS tape caused by the heat transfer of the nano-silver paste. The optimal heating time was about one minute for our study.

The heating temperature, applied pressure, specific resistance, and process time for different studies are tabulated in Table 2 for the purpose of comparison. Using the Sn-alloy as solder shows good specific resistance for most studies (0.01–0.047 μΩ·cm^2^), which was better than our study. However, the Sn-alloy formed Cu_6_Sn_5_ and Cu_3_Sn IMC (intermetallic compound) on the cupper-solder interface at a temperature as low as 125 °C. The formation of IMC reduces the reliability of joints used in high power electronics for operations carried out over long time periods [32]. Most studies [9,10,33] have reported that the HTS joining process using Sn-alloy solder is heated at 190–230 °C, which means that the IMC will form at the solder–Cu interface. The use of the indium foil as solder has shown relatively low specific resistance; however, this may be due to the high compressive stress of about 100 MPa that was used [11]. Nakanishi et al. [13] reported using sintered metal paste (Au, Ag) as the solder material similar to this study, but with the furnace heating method. The reported similar unstable superconducting properties to those we reported in this study.

## 5. Conclusions

This study used nano-silver paste as the solder material of the 2G HTS tape joint. Two types of heating methods, i.e., tube furnace heating and resistive Joule heating, were used to sinter nano-silver paste. For the furnace heating method (heat flux outside-in), it was concluded that the most appropriate heating temperature was 200–215 °C, which showed the joint specific resistance of 0.2–0.3 μΩ∙cm^2^, and was similar to the traditional Sn-alloy; the *I_c_* retention percentage was only 85%. For the resistive Joule heating method (heat flux inside-out), the high resistance of the joint can produce a significant heat concentration effect, which successfully sintered the nano-silver paste at the 2G HTS tape joint. The specific resistance of the best sample was 0.074 μΩ∙cm^2^; the *I_c_* retention percentage was 99%. This result indicates that the internal heat flux shows less deteriorating impact on the 2G RE-Ba-Cu-O tape during the sintering process of the nano-silver paste at the joint. That is, for application such as coil joints instead of the furnace heating method, the resistive Joule heating method can easily achieve low specific joint resistance without affecting the performance of the entire coils.

## Figures and Tables

**Table 1:** Superconducting properties of nano-silver paste joining HTS tape samples: heating temperature, *I_c_* retention percentage, and specific resistance.

Process	Sample Name	Heating Temperature (°C)	*I_c_* Before Joining (A)	*I_c_* After Joining (A)	*I_c_* Retention Percentage	Specific Resistance (μΩ∙cm^2^)
Furnace Heating Sample	Furnace 1	180	99.4	−	−	948
	Furnace 2	200	90.6	83.2	92%	1.03
	Furnace 3	215	97.5	83.2	85%	0.21
	Furnace 4	230	94.5	69.9	72%	0.20
	Furnace 5	250	97.6	42.7	44%	0.29
Resistive Joule Heating Sample	Joule 1	103	92.5	77.6	84%	0.697
	Joule 2	116.2	89.4	87.8	98%	0.468
	Joule 3	156	101.3	100.0	99%	0.054~0.074
	Joule 4	177.4	91.8	86.6	94%	0.686
	Joule 5	228.6	86.9	78.0	90%	15.0

**Table 2:** The heating temperature, applied pressure, specific resistance, and process time for different studies.

Solder Material	Heating Temperature	Specific Resistance	Reference
Sn_36_Pb_37_	190 °C	0.025~0.027 μΩ·cm^2^	[9]
Pb_39_Sn_61_	200 °C (Lamination joint)	0.010~0.015 μΩ·cm^2^	[10]
In	90~140 °C	~0.025 μΩ·cm^2^	[11]
In	Ultrasonic welding	0.031~0.032 μΩ·cm^2^	[12]
Sn_96.5_Ag_3_Cu_0.5_	226~233 °C	0.067~0.070 μΩ·cm^2^	[33]
Nano metal paste	150~200 °C	~0.048 μΩ·cm^2^	[13]
Nano-silver paste	150~200 °C	0.054~15 μΩ·cm^2^	this study

## Data Availability

Not applicable.

## References

[1] Lvovsky Y., Stautner E.W., Zhang T. (2013). Novel technologies and configurations of superconducting magnets for MRI. Supercond. Sci. Technol..

[2] Yanagisawa Y., Piao R., Iguchi S., Nakagome H., Takao T., Kominato K., Hamada M., Matsumoto S., Suematsu H., Jin X. (2014). Operation of a 400 MHz NMR magnet using a (RE: Rare earth) Ba_2_Cu_3_O_7_− x high-temperature superconducting coil: Towards an ultra-compact super-high field NMR spectrometer operated beyond 1 GHz. J. Magn. Reson..

[3] Uglietti D. (2019). A review of commercial high temperature superconducting materials for large magnets: From wires and tapes to cables and conductors. Supercond. Sci. Technol..

[4] Martucciello N., Giubileo F., Grimaldi G., Corato V. (2015). Introduction to the focus on superconductivity for energy. Supercond. Sci. Technol..

[5] Selvamanickam V., Chen Y., Xiong X., Xie Y., Zhang X., Rar A., Martchevskii M., Schmidt R., Lenseth K., Herrin J. (2008). Progress in second-generation HTS wire development and manufacturing. Phys. C Supercond..

[6] Larbalestier D., Gurevich A., Feldmann D.M., Polyanskii A. (2011). High-Tc superconducting materials for electric power applications. Mater. Sustain. Energy A Collect. Peer-Rev. Res. Rev. Artic. Nat. Publ. Group.

[7] Gurevich A. (2011). To use or not to use cool superconductors?. Nat. Mater..

[8] Tsui Y., Surrey E., Hampshire D. (2016). Soldered joints—an essential component of demountable high temperature superconducting fusion magnets. Supercond. Sci. Technol..

[9] Huang D., Gu H., Dong Z., Shang H., Xu W., Li T., Xie B., Zhang H., Ding F. (2019). Study on electromechanical properties of solder jointed YBCO coated conductors with etched copper stabilizer under axial tension. IEEE Trans. Appl. Supercond..

[10] Balashov N.N., Degtyarenko P.N., Ivanov S.S., Kopylov S.I., Gorbunova D.A., Molodyk A.A., Samoilenkov S.V., Sytnikov V.E., Zheltov V.V. (2018). Low-resistance soldered joints of commercial 2G HTS wire prepared at various values of applied pressure. IEEE Trans. Appl. Supercond..

[11] Nishio T., Ito S., Hashizume H. (2017). Heating and loading process improvement for indium inserted mechanical lap joint of REBCO tapes. IEEE Trans. Appl. Supercond..

[12] Ito S., Fujii H.T., Hayasaka R., Sato Y.S., Hashizume H. (2019). Comparison of heat assisted lap joints of high-temperature superconducting tapes with inserted indium foils. IEEE Trans. Appl. Supercond..

[13] Nakanishi T., Machi T., Izumi T., Teranishi R., Kato T., Kato T., Hirayama T. (2016). Jointing of coated conductors by using nano-particle metal pastes. Phys. Procedia.

[14] Kato J., Sakai N., Miyata S., Konishi M., Yamada Y., Chikumoto N., Nakao K., Izumi T., Shiohara Y. (2007). Optimization of the diffusion joint process for the Ag layers of YBCO coated conductors. Phys. C Supercond. Its Appl..

[15] Kato J., Sakai N., Miyata S., Ibi A., Sutoh Y., Yamada Y., Chikumoto N., Nakao K., Izumi T., Shiohara Y. (2008). Diffusion joint using silver layer of YBCO coated conductors for applications. Phys. C Supercond..

[16] Park Y., Lee M., Ann H., Choi Y.H., Lee H. (2014). A superconducting joint for GdBa_2_Cu_3_O_7_− δ-coated conductors. NPG Asia Mater..

[17] Jin X., Yanagisawa Y., Maeda H., Takano Y. (2015). Development of a superconducting joint between a GdBa_2_Cu_3_O_7_-δ-coated conductor and YBa_2_Cu_3_O_7_− δ bulk: Towards a superconducting joint between RE (Rare Earth) Ba_2_Cu_3_O_7_− δ-coated conductors. Supercond. Sci. Technol..

[18] Jin X., Yanagisawa Y., Maeda H. (2018). Measurement of persistent current in a Gd123 coil with a superconducting joint fabricated by the CJMB method. IEEE Trans. Appl. Supercond..

[19] Ohki K., Nagaishi T., Kato T., Yokoe D., Hirayama T., Ikuhara Y., Ueno T., Yamagishi K., Takao T., Piao R. (2017). Fabrication, microstructure and persistent current measurement of an intermediate grown superconducting (iGS) joint between REBCO-coated conductors. Supercond. Sci. Technol..

[20] Yazaki S., Karasawa A., Kotoyori T., Ishiyama A., Miyahara N. (2013). Critical current degradation in high-temperature superconducting tapes caused by temperature rise. IEEE Trans. Appl. Supercond..

[21] Preuss A., Fietz W.H., Immel F., Kauffmann-Weiss S., Wolf M.J. (2018). Critical current degradation of coated conductors under soldering conditions. IEEE Trans. Appl. Supercond..

[22] Mei Y., Chen G., Cao Y., Li X., Han D., Chen X. (2013). Simplification of low-temperature sintering nanosilver for power electronics packaging. J. Electron. Mater..

[23] Mei Y., Li L., Li X., Li W., Yan H., Xie Y. (2017). Electric-current-assisted sintering of nanosilver paste for copper bonding. J. Mater. Sci. : Mater. Electron..

[24] Mei Y.-H., Cao Y., Chen G., Li X., Lu G.-Q., Chen X. (2013). Characterization and reliability of sintered nanosilver joints by a rapid current-assisted method for power electronics packaging. IEEE Trans. Device Mater. Reliab..

[25] Kim Y.-J., Park B.-H., Hyun S.-K., Nishikawa H. (2021). The influence of porosity and pore shape on the thermal conductivity of silver sintered joint for die attach. Mater. Today Commun..

[26] Hsu S.L.-C., Chen Y.-T., Chen M.-L., Chen I.-G. (2021). Low Sintering Temperature Nano-Silver Pastes with High Bonding Strength by Adding Silver 2-Ethylhexanoate. Materials.

[27] Skarba M., Pekarčíková M., Frolek L., Cuninková E., Necpal M. (2021). Thermal Cycling of (RE) BCO-Based Superconducting Tapes Joined by Lead-Free Solders. Materials.

[28] Ito S., Tamura H., Yanagi N., Hashizume H. (2021). Low-resistance joint development for segment-fabrication of high-temperature superconducting fusion magnets. Nucl. Fusion.

[29] Murtomäki J.S., Kirby G., van Nugteren J., Contat P.-A., Sacristan-de-Frutos O., Fleiter J., Pincot F.-O., de Rijk G., Rossi L., Ruuskanen J. (2018). 10 kA joints for HTS Roebel cables. IEEE Trans. Appl. Supercond..

[30] Gao S., Yang Z., Tan Y., Li X., Chen X., Sun Z., Lu G.-Q. (2018). Bonding of large substrates by silver sintering and characterization of the interface thermal resistance. IEEE Trans. Ind. Appl..

[31] Dai J., Li J., Agyakwa P., Corfield M., Johnson C.M. (2018). Comparative thermal and structural characterization of sintered nano-silver and high-lead solder die attachments during power cycling. IEEE Trans. Device Mater. Reliab..

[32] Lee T., Choi W., Tu K.-N., Jang J., Kuo S., Lin J., Frear D., Zeng K., Kivilahti J. (2002). Morphology, kinetics, and thermodynamics of solid-state aging of eutectic SnPb and Pb-free solders (Sn–3.5 Ag, Sn–3.8 Ag–0.7 Cu and Sn–0.7 Cu) on Cu. J. Mater. Res..

[33] Michalcová E., Gömöry F., Frolek L., Drienovský M., Pekarčíková M., Skarba M., Mišík J., Janovec J. (2016). Joining of CC tapes with lead-free solders. IEEE Trans. Appl. Supercond..

